# Dichlorido(η^6^-*p*-cymene)(eth­oxy­diphenyl­phosphane)ruthenium(II)

**DOI:** 10.1107/S1600536812045461

**Published:** 2012-11-10

**Authors:** Spring M. M. Knapp, Lev N. Zakharov, David R. Tyler

**Affiliations:** a Department of Chemistry, 1253 University of Oregon, Eugene, Oregon 97403-1253, USA

## Abstract

The title compound, [RuCl_2_(C_10_H_14_)(C_14_H_15_OP)], is an Ru^II^ complex in which an η^6^-*p*-cymene ligand, two chloride anions and the P atom of an ethoxydiphenylphosphane ligand form a piano-stool coordination environment about the central Ru^II^ atom.

## Related literature

For related structures [Ru(η^6^-*p*-cymene)Cl_2_PPh_3_] and [Ru(η^6^-*p*-cymene)Cl_2_PPhOEt_2_], see: Elsegood *et al.* (2006 ▶) and Alber­tin *et al.* (2010 ▶), respectively. For the application of similar complexes as nitrile hydration catalysts, see: Ahmed *et al.* (2009 ▶); Cavarzan *et al.* (2010 ▶); Cadierno *et al.* (2008 ▶); García-Álvarez *et al.* (2010 ▶, 2011 ▶); Knapp *et al.* (2012 ▶).
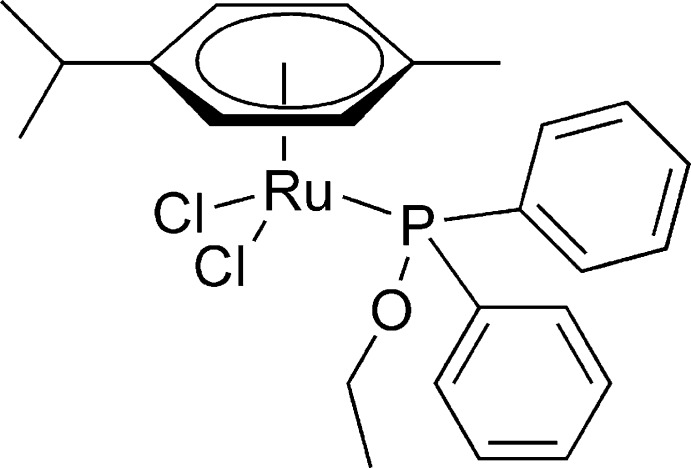


## Experimental

### 

#### Crystal data

[RuCl_2_(C_10_H_14_)(C_14_H_15_OP)]
*M* *_r_* = 536.41
Monoclinic, 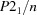

*a* = 13.1818 (7) Å
*b* = 10.8481 (6) Å
*c* = 16.6888 (9) Å
β = 95.060 (1)°
*V* = 2377.2 (2) Å^3^
*Z* = 4
Mo *K*α radiation
μ = 0.97 mm^−1^
*T* = 173 K
0.23 × 0.18 × 0.06 mm

#### Data collection

Bruker APEX CCD area-detector diffractometer
Absorption correction: multi-scan (*SADABS*; Sheldrick, 1995 ▶) *T*_min_ = 0.809, *T*_max_ = 0.944
15739 measured reflections
5164 independent reflections
4294 reflections with *I* > 2σ(*I*)
*R*_int_ = 0.028

#### Refinement

*R*[*F* ^2^ > 2σ(*F* ^2^)] = 0.030
*wR*(*F* ^2^) = 0.068
*S* = 1.05
5164 reflections
378 parameters
All H-atom parameters refined
Δρ_max_ = 0.50 e Å^−3^
Δρ_min_ = −0.47 e Å^−3^

### 

Data collection: *SMART* (Bruker, 2000 ▶); cell refinement: *SAINT* (Bruker, 2000 ▶); data reduction: *SAINT*; program(s) used to solve structure: *SHELXTL* (Sheldrick, 2008 ▶); program(s) used to refine structure: *SHELXTL*; molecular graphics: *SHELXTL*; software used to prepare material for publication: *SHELXTL*.

## Supplementary Material

Crystal structure: contains datablock(s) I, global. DOI: 10.1107/S1600536812045461/sj5274sup1.cif

Structure factors: contains datablock(s) I. DOI: 10.1107/S1600536812045461/sj5274Isup2.hkl

Additional supplementary materials:  crystallographic information; 3D view; checkCIF report

## Figures and Tables

**Table 1:** Selected geometric parameters (Å, °) for the title compound and related compounds Σ angles = sum of P—Ru—Cl1, P—Ru—Cl2, and Cl1—Ru— Cl2 angles.

	Title compound	[Ru(η^6^-*p*-cymene)Cl_2_PPhOEt_2_]*^*a*^*	[Ru(η^6^-*p*-cymene)Cl_2_PPh_3_]*^*b*^*
Ru—P	2.3147 (6)	2.2807 (7)	2.3438 (6)
Ru—Cl1	2.4124 (6)	2.4171 (7)	2.4154 (6)
Ru—Cl2	2.3992 (6)	2.4038 (7)	2.4151 (6)
Ru—C(av)	2.217 (1)	2.218 (4)	2.218 (2)
P—Ru – Cl1	90.67 (2)	87.59 (2)	87.094 (19)
P—Ru – Cl2	84.75 (2)	87.89 (2)	90.27 (2)
Cl1—Ru – Cl2	90.04 (2)	88.81 (2)	88.41 (2)
Σ angles	265.46	264.29	265.77

Notes: (*a*) Albertin *et al.* (2010 ▶); (*b*) Elsegood *et al.* (2006 ▶).

**Fig. 1.**
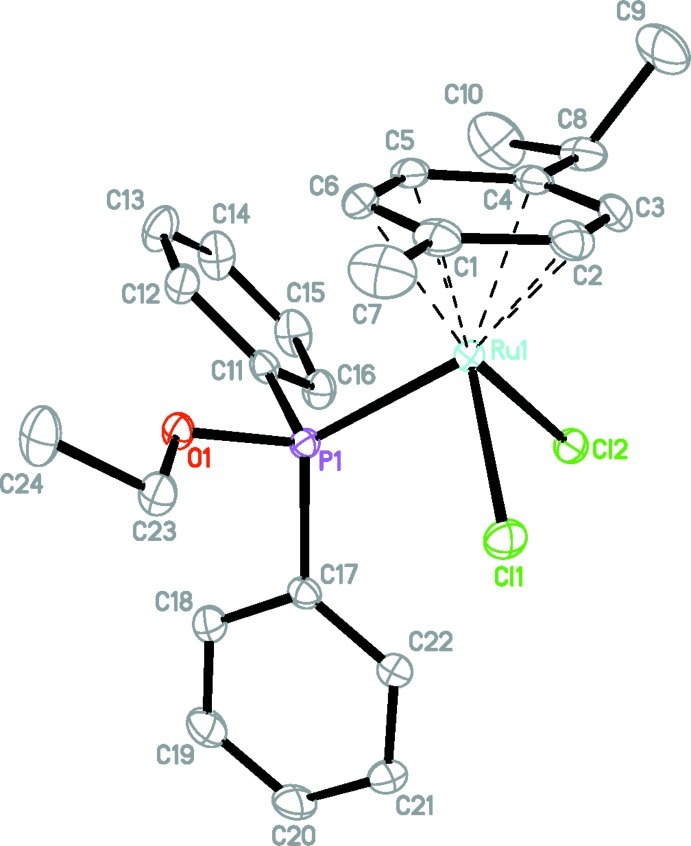
The crystal structure of dichloro(η6-p-cymene)(ethoxydiphenylphosphane)ruthenium(II) with 50% probability displacement ellipsoids and the atom-numbering scheme. H atoms are omitted for clarity.

## supplementary crystallographic information

### Comment

Investigations in our laboratory have focused on the synthesis and study of nitrile hydration catalysts for the hydration of cyanohydrins (Ahmed *et al.*, 2009). Application of other similar complexes as nitrile hydration catalysts have also been reported (Cavarzan, *et al.*, 2010; Cadierno, *et al.*, 2008; García-Álvarez, *et al.*, 2010, 2011; Knapp, *et al.*, 2012). These catalysts have been found to be susceptible to poisoning by cyanide, which forms when cyanohydrins decompose in aqueous solutions. The title compound [Ru(*η*^6^-*p*-cymene)Cl_2_PPh_2_OEt] (1), as well as the similar compounds [Ru(*η*^6^-*p*-cymene)Cl_2_PPhOEt_2_], (Albertin, *et al.*, 2010), (2), and [Ru(*η*^6^-*p*- cymene)Cl_2_PPh_3_] (3), (Elsegood, *et al.*, 2006), have been used previously to hydrate nitriles in aqueous solutions, where addition of surfactant was used to increase the solubility of the catalyst by promoting the formation of micelles (Cavarzan, *et al.*, 2010). It was hypothesized that a hydrophobic catalyst would be resistant to cyanide poisoning under biphasic conditions, because the cyanide would be in the aqueous phase and the catalyst in the organic phase. Therefore, 1 was synthesized and used as a nitrile hydration catalyst under biphasic conditions using 1,1,2,2-tetrachloroethane at 100 °C. Hydration of the model nitriles acetonitrile and 3-hydroxypropionitrile went to completion within 48 h under these conditions. Unfortunately, no hydration of the cyanohydrins glycolonitrile, lactonitrile, or acetone cyanohydrin was observed.

The structure of complex 1 reported here adopts the classic piano stool structure with a pseudo-tetrahedral arrangement of the *p*-cymene, chloride anions and the phosphane about the ruthenium metal center. The bond lengths and angles about the Ru core compare well with 2 (Albertin, *et al.*, 2010) and 3 (Elsegood, *et al.*, 2006), which have been investigated previously (Table 1). The average Ru – C distance is 2.217 (3) Å, which is very similar to the previously reported Ru – C distances of 2.218 (4) Å and 2.218 (2) Å for 2 and 3, respectively. The Ru – C bonds *trans* to the phosphane are lengthened, as has been observed previously (Elsegood, *et al.*, 2006), where Ru – C2 (2.228 (3) Å) and Ru – C3 (2.242 (2) Å) are longer than the other Ru – C bonds (average length 2.208 (3) Å). A comparison of the sum of the P – Ru – Cl1, P – Ru – Cl2, and Cl1 – Ru – Cl2 angles between 1 – 3 indicates that, as expected, 1 is more sterically hindered than 2 and less hindered than 3 (Table 1).

### Experimental

The title compound was prepared following literature procedures (Albertin, *et al*., 2010). The red solid was dissolved in THF under a nitrogen atmosphere, and single crystals were obtained by slow evaporation of THF.

### Refinement

H atoms were found on the residual density and refined with isotropic thermal parameters.

### Crystal data

**Table:**

[RuCl_2_(C_10_H_14_)(C_14_H_15_OP)]	*F*(000) = 1096
*M**_r_* = 536.41	*D*_x_ = 1.499 Mg m^−^^3^
Monoclinic, *P*2_1_/*n*	Mo *K*α radiation, λ = 0.71073 Å
Hall symbol: -P 2yn	Cell parameters from 4833 reflections
*a* = 13.1818 (7) Å	θ = 2.2–28.2°
*b* = 10.8481 (6) Å	µ = 0.97 mm^−^^1^
*c* = 16.6888 (9) Å	*T* = 173 K
β = 95.060 (1)°	Plate, red
*V* = 2377.2 (2) Å^3^	0.23 × 0.18 × 0.06 mm
*Z* = 4	

### Data collection

**Table:**

Bruker APEX CCD area-detector diffractometer	5164 independent reflections
Radiation source: fine-focus sealed tube	4294 reflections with *I* > 2σ(*I*)
Graphite monochromator	*R*_int_ = 0.028
φ and ω scans	θ_max_ = 27.0°, θ_min_ = 1.9°
Absorption correction: multi-scan (*SADABS*; Sheldrick, 1995)	*h* = −12→16
*T*_min_ = 0.809, *T*_max_ = 0.944	*k* = −13→12
15739 measured reflections	*l* = −20→21

### Refinement

**Table:**

Refinement on *F*^2^	Primary atom site location: structure-invariant direct methods
Least-squares matrix: full	Secondary atom site location: difference Fourier map
*R*[*F*^2^ > 2σ(*F*^2^)] = 0.030	Hydrogen site location: difference Fourier map
*wR*(*F*^2^) = 0.068	All H-atom parameters refined
*S* = 1.05	*w* = 1/[σ^2^(*F*_o_^2^) + (0.0316*P*)^2^ + 0.5053*P*] where *P* = (*F*_o_^2^ + 2*F*_c_^2^)/3
5164 reflections	(Δ/σ)_max_ = 0.002
378 parameters	Δρ_max_ = 0.50 e Å^−^^3^
0 restraints	Δρ_min_ = −0.47 e Å^−^^3^

### Special details

**Table:**

Geometry. All e.s.d.'s (except the e.s.d. in the dihedral angle between two l.s. planes)are estimated using the full covariance matrix. The cell e.s.d.'s are takeninto account individually in the estimation of e.s.d.'s in distances, anglesand torsion angles; correlations between e.s.d.'s in cell parameters are onlyused when they are defined by crystal symmetry. An approximate (isotropic)treatment of cell e.s.d.'s is used for estimating e.s.d.'s involving l.s. planes.
Refinement. Refinement of *F*^2^ against ALL reflections. The weighted *R*-factor *wR* andgoodness of fit *S* are based on *F*^2^, conventional *R*-factors *R* are basedon *F*, with *F* set to zero for negative *F*^2^. The threshold expression of*F*^2^ > σ(*F*^2^) is used only for calculating *R*-factors(gt) *etc*. and isnot relevant to the choice of reflections for refinement. *R*-factors basedon *F*^2^ are statistically about twice as large as those based on *F*, and *R*-factors based on ALL data will be even larger.

### Fractional atomic coordinates and isotropic or equivalent isotropic displacement parameters (Å^2^)

**Table:**

	*x*	*y*	*z*	*U*_iso_*/*U*_eq_	
Ru1	0.922759 (14)	0.993847 (17)	0.179893 (11)	0.02079 (7)	
Cl1	0.85769 (5)	1.20143 (6)	0.16713 (4)	0.03260 (15)	
Cl2	1.08699 (5)	1.06510 (6)	0.14859 (4)	0.02962 (14)	
P1	0.98348 (5)	1.03105 (5)	0.31207 (4)	0.02006 (13)	
O1	0.90032 (12)	1.01438 (14)	0.37705 (10)	0.0244 (4)	
C1	0.76577 (19)	0.9210 (2)	0.15958 (17)	0.0346 (6)	
C2	0.8041 (2)	0.9389 (3)	0.08317 (16)	0.0347 (6)	
C3	0.8954 (2)	0.8889 (3)	0.06431 (16)	0.0335 (6)	
C4	0.95485 (19)	0.8142 (2)	0.12134 (15)	0.0292 (6)	
C5	0.9169 (2)	0.7927 (2)	0.19614 (16)	0.0287 (6)	
C6	0.8235 (2)	0.8463 (2)	0.21485 (16)	0.0307 (6)	
C7	0.6666 (3)	0.9770 (4)	0.1789 (3)	0.0537 (9)	
C8	1.0545 (2)	0.7606 (3)	0.09785 (19)	0.0407 (7)	
C9	1.0331 (3)	0.6736 (4)	0.0262 (3)	0.0617 (10)	
C10	1.1180 (3)	0.6994 (4)	0.1653 (3)	0.0686 (12)	
C11	1.07110 (18)	0.9129 (2)	0.35063 (14)	0.0245 (5)	
C12	1.0370 (2)	0.8121 (2)	0.39239 (15)	0.0319 (6)	
C13	1.1040 (2)	0.7190 (3)	0.41857 (18)	0.0444 (8)	
C14	1.2050 (2)	0.7263 (3)	0.40394 (18)	0.0446 (8)	
C15	1.2399 (2)	0.8264 (3)	0.36299 (17)	0.0403 (7)	
C16	1.1739 (2)	0.9189 (3)	0.33660 (16)	0.0327 (6)	
C17	1.05088 (17)	1.1723 (2)	0.34448 (14)	0.0238 (5)	
C18	1.0912 (2)	1.1768 (3)	0.42473 (16)	0.0336 (6)	
C19	1.1478 (2)	1.2776 (3)	0.45319 (18)	0.0447 (7)	
C20	1.1628 (2)	1.3750 (3)	0.40183 (19)	0.0448 (8)	
C21	1.1217 (2)	1.3722 (3)	0.32335 (18)	0.0384 (7)	
C22	1.0657 (2)	1.2705 (2)	0.29405 (16)	0.0297 (6)	
C23	0.8140 (2)	1.0977 (3)	0.37466 (17)	0.0317 (6)	
C24	0.7362 (2)	1.0397 (3)	0.4228 (2)	0.0446 (8)	
H2	0.770 (2)	0.993 (2)	0.0517 (17)	0.033 (8)*	
H3	0.9204 (18)	0.910 (2)	0.0173 (15)	0.029 (7)*	
H5	0.956 (2)	0.751 (3)	0.2361 (16)	0.038 (8)*	
H6	0.8040 (18)	0.836 (2)	0.2656 (15)	0.027 (7)*	
H7A	0.612 (3)	0.942 (3)	0.147 (2)	0.065 (11)*	
H7B	0.649 (3)	0.982 (3)	0.234 (3)	0.093 (15)*	
H7C	0.667 (2)	1.058 (3)	0.1647 (19)	0.053 (10)*	
H8	1.092 (2)	0.827 (3)	0.0773 (19)	0.059 (10)*	
H9A	0.991 (3)	0.704 (4)	−0.019 (3)	0.109 (18)*	
H9B	0.997 (3)	0.593 (4)	0.046 (2)	0.091 (13)*	
H9C	1.100 (3)	0.650 (3)	0.010 (2)	0.072 (11)*	
H10A	1.179 (3)	0.667 (3)	0.148 (2)	0.072 (11)*	
H10B	1.129 (3)	0.747 (4)	0.213 (3)	0.100 (15)*	
H10C	1.077 (2)	0.625 (3)	0.1826 (18)	0.051 (10)*	
H12	0.964 (2)	0.806 (2)	0.4018 (15)	0.036 (7)*	
H13	1.0801 (19)	0.650 (2)	0.4488 (15)	0.033 (7)*	
H14	1.249 (2)	0.662 (3)	0.4195 (17)	0.047 (8)*	
H15	1.309 (2)	0.828 (3)	0.3514 (16)	0.042 (8)*	
H16	1.192 (2)	0.985 (2)	0.3115 (16)	0.029 (7)*	
H18	1.0840 (19)	1.115 (3)	0.4602 (16)	0.036 (8)*	
H19	1.175 (2)	1.280 (3)	0.5067 (18)	0.052 (9)*	
H20	1.202 (2)	1.435 (3)	0.4187 (16)	0.038 (8)*	
H21	1.133 (2)	1.438 (3)	0.2895 (18)	0.050 (9)*	
H22	1.0405 (18)	1.269 (2)	0.2397 (15)	0.026 (7)*	
H23A	0.790 (2)	1.104 (3)	0.3202 (18)	0.051 (9)*	
H23B	0.8364 (19)	1.178 (3)	0.3987 (15)	0.034 (7)*	
H24A	0.709 (3)	0.965 (3)	0.396 (2)	0.063 (11)*	
H24B	0.762 (3)	1.026 (3)	0.479 (2)	0.064 (11)*	
H24C	0.681 (3)	1.094 (3)	0.4211 (19)	0.067 (11)*	

### Atomic displacement parameters (Å^2^)

**Table:**

	*U*^11^	*U*^22^	*U*^33^	*U*^12^	*U*^13^	*U*^23^
Ru1	0.02214 (11)	0.02052 (11)	0.01992 (10)	−0.00193 (8)	0.00301 (7)	0.00024 (7)
Cl1	0.0357 (4)	0.0261 (3)	0.0359 (3)	0.0058 (3)	0.0025 (3)	0.0038 (3)
Cl2	0.0299 (3)	0.0306 (3)	0.0299 (3)	−0.0086 (3)	0.0110 (3)	−0.0030 (3)
P1	0.0208 (3)	0.0191 (3)	0.0207 (3)	0.0008 (2)	0.0042 (2)	0.0004 (2)
O1	0.0241 (9)	0.0250 (9)	0.0251 (9)	0.0060 (7)	0.0082 (7)	0.0024 (7)
C1	0.0259 (14)	0.0330 (15)	0.0448 (16)	−0.0073 (11)	0.0019 (12)	−0.0089 (12)
C2	0.0365 (16)	0.0329 (15)	0.0321 (15)	−0.0023 (12)	−0.0104 (12)	−0.0027 (12)
C3	0.0435 (16)	0.0358 (15)	0.0214 (13)	−0.0113 (12)	0.0046 (12)	−0.0073 (11)
C4	0.0293 (14)	0.0235 (13)	0.0357 (14)	−0.0078 (10)	0.0072 (11)	−0.0098 (11)
C5	0.0335 (15)	0.0207 (13)	0.0312 (14)	−0.0063 (10)	−0.0008 (12)	−0.0019 (10)
C6	0.0343 (15)	0.0290 (14)	0.0302 (14)	−0.0125 (11)	0.0103 (12)	−0.0018 (11)
C7	0.0286 (17)	0.055 (2)	0.078 (3)	−0.0012 (15)	0.0034 (18)	−0.013 (2)
C8	0.0367 (16)	0.0306 (15)	0.0564 (19)	−0.0051 (13)	0.0132 (14)	−0.0133 (14)
C9	0.060 (2)	0.067 (3)	0.062 (2)	0.002 (2)	0.025 (2)	−0.030 (2)
C10	0.058 (3)	0.074 (3)	0.075 (3)	0.030 (2)	0.008 (2)	−0.018 (2)
C11	0.0262 (13)	0.0260 (13)	0.0215 (12)	0.0047 (10)	0.0043 (10)	−0.0031 (10)
C12	0.0340 (15)	0.0308 (14)	0.0319 (14)	0.0081 (11)	0.0091 (12)	0.0053 (11)
C13	0.0538 (19)	0.0371 (17)	0.0446 (18)	0.0145 (14)	0.0173 (15)	0.0175 (14)
C14	0.0479 (19)	0.0487 (19)	0.0380 (16)	0.0287 (15)	0.0087 (14)	0.0106 (14)
C15	0.0295 (15)	0.0545 (19)	0.0377 (16)	0.0183 (13)	0.0068 (13)	0.0021 (14)
C16	0.0306 (15)	0.0373 (16)	0.0307 (14)	0.0054 (12)	0.0060 (11)	0.0030 (12)
C17	0.0221 (12)	0.0241 (12)	0.0256 (12)	0.0005 (10)	0.0047 (10)	−0.0040 (10)
C18	0.0402 (16)	0.0344 (15)	0.0263 (13)	−0.0053 (12)	0.0039 (12)	−0.0008 (12)
C19	0.056 (2)	0.0455 (18)	0.0320 (16)	−0.0122 (15)	0.0005 (14)	−0.0122 (14)
C20	0.0526 (19)	0.0357 (17)	0.0466 (18)	−0.0199 (15)	0.0068 (15)	−0.0145 (14)
C21	0.0488 (18)	0.0270 (15)	0.0405 (16)	−0.0090 (13)	0.0107 (13)	−0.0018 (12)
C22	0.0351 (15)	0.0269 (14)	0.0272 (13)	−0.0018 (11)	0.0025 (11)	−0.0022 (11)
C23	0.0311 (15)	0.0308 (15)	0.0346 (15)	0.0120 (11)	0.0121 (12)	0.0054 (12)
C24	0.0333 (17)	0.059 (2)	0.0442 (19)	0.0145 (15)	0.0171 (14)	0.0121 (16)

### Geometric parameters (Å, º)

**Table:**

Ru1—C6	2.180 (2)	C9—H9C	0.98 (4)
Ru1—C5	2.201 (2)	C10—H10A	0.95 (4)
Ru1—C1	2.213 (2)	C10—H10B	0.95 (4)
Ru1—C2	2.228 (3)	C10—H10C	1.03 (3)
Ru1—C4	2.237 (2)	C11—C12	1.393 (3)
Ru1—C3	2.242 (2)	C11—C16	1.397 (3)
Ru1—P1	2.3147 (6)	C12—C13	1.386 (4)
Ru1—Cl2	2.3992 (6)	C12—H12	0.98 (3)
Ru1—Cl1	2.4124 (6)	C13—C14	1.377 (4)
P1—O1	1.6187 (17)	C13—H13	0.97 (3)
P1—C11	1.805 (2)	C14—C15	1.383 (4)
P1—C17	1.829 (2)	C14—H14	0.92 (3)
O1—C23	1.451 (3)	C15—C16	1.375 (4)
C1—C6	1.401 (4)	C15—H15	0.95 (3)
C1—C2	1.426 (4)	C16—H16	0.87 (3)
C1—C7	1.502 (4)	C17—C22	1.382 (3)
C2—C3	1.382 (4)	C17—C18	1.398 (3)
C2—H2	0.88 (3)	C18—C19	1.383 (4)
C3—C4	1.430 (4)	C18—H18	0.91 (3)
C3—H3	0.91 (2)	C19—C20	1.386 (4)
C4—C5	1.404 (3)	C19—H19	0.93 (3)
C4—C8	1.519 (4)	C20—C21	1.373 (4)
C5—C6	1.422 (4)	C20—H20	0.86 (3)
C5—H5	0.92 (3)	C21—C22	1.391 (4)
C6—H6	0.91 (2)	C21—H21	0.93 (3)
C7—H7A	0.94 (4)	C22—H22	0.94 (2)
C7—H7B	0.98 (4)	C23—C24	1.497 (4)
C7—H7C	0.91 (3)	C23—H23A	0.94 (3)
C8—C10	1.497 (5)	C23—H23B	1.00 (3)
C8—C9	1.530 (4)	C24—H24A	0.98 (3)
C8—H8	0.96 (3)	C24—H24B	0.98 (4)
C9—H9A	0.95 (4)	C24—H24C	0.94 (3)
C9—H9B	1.06 (4)		
			
C6—Ru1—C5	37.88 (10)	C1—C6—H6	120.1 (16)
C6—Ru1—C1	37.18 (10)	C5—C6—H6	118.3 (16)
C5—Ru1—C1	67.83 (10)	Ru1—C6—H6	124.3 (16)
C6—Ru1—C2	66.27 (10)	C1—C7—H7A	110 (2)
C5—Ru1—C2	78.12 (10)	C1—C7—H7B	121 (3)
C1—Ru1—C2	37.45 (10)	H7A—C7—H7B	109 (3)
C6—Ru1—C4	67.57 (9)	C1—C7—H7C	108 (2)
C5—Ru1—C4	36.88 (9)	H7A—C7—H7C	106 (3)
C1—Ru1—C4	80.35 (9)	H7B—C7—H7C	102 (3)
C2—Ru1—C4	66.31 (10)	C10—C8—C4	114.5 (3)
C6—Ru1—C3	78.61 (10)	C10—C8—C9	111.5 (3)
C5—Ru1—C3	66.28 (10)	C4—C8—C9	109.5 (3)
C1—Ru1—C3	67.02 (10)	C10—C8—H8	109.3 (19)
C2—Ru1—C3	36.02 (10)	C4—C8—H8	106.9 (19)
C4—Ru1—C3	37.24 (10)	C9—C8—H8	104.6 (19)
C6—Ru1—P1	92.19 (7)	C8—C9—H9A	117 (3)
C5—Ru1—P1	93.92 (7)	C8—C9—H9B	109 (2)
C1—Ru1—P1	116.63 (7)	H9A—C9—H9B	106 (3)
C2—Ru1—P1	154.01 (8)	C8—C9—H9C	106 (2)
C4—Ru1—P1	120.35 (7)	H9A—C9—H9C	110 (3)
C3—Ru1—P1	157.59 (8)	H9B—C9—H9C	108 (3)
C6—Ru1—Cl2	150.41 (8)	C8—C10—H10A	112 (2)
C5—Ru1—Cl2	112.84 (7)	C8—C10—H10B	115 (3)
C1—Ru1—Cl2	158.61 (7)	H10A—C10—H10B	112 (3)
C2—Ru1—Cl2	121.18 (8)	C8—C10—H10C	106.6 (17)
C4—Ru1—Cl2	88.65 (6)	H10A—C10—H10C	106 (3)
C3—Ru1—Cl2	93.21 (7)	H10B—C10—H10C	104 (3)
P1—Ru1—Cl2	84.75 (2)	C12—C11—C16	118.7 (2)
C6—Ru1—Cl1	119.46 (7)	C12—C11—P1	120.77 (18)
C5—Ru1—Cl1	156.97 (7)	C16—C11—P1	120.46 (19)
C1—Ru1—Cl1	89.97 (7)	C13—C12—C11	120.4 (3)
C2—Ru1—Cl1	87.95 (8)	C13—C12—H12	120.1 (16)
C4—Ru1—Cl1	148.65 (7)	C11—C12—H12	119.5 (16)
C3—Ru1—Cl1	111.67 (8)	C14—C13—C12	120.0 (3)
P1—Ru1—Cl1	90.67 (2)	C14—C13—H13	120.1 (15)
Cl2—Ru1—Cl1	90.04 (2)	C12—C13—H13	119.8 (15)
O1—P1—C11	97.57 (10)	C13—C14—C15	120.2 (3)
O1—P1—C17	103.62 (10)	C13—C14—H14	119.6 (18)
C11—P1—C17	102.15 (11)	C15—C14—H14	120.2 (18)
O1—P1—Ru1	114.92 (7)	C16—C15—C14	120.1 (3)
C11—P1—Ru1	111.87 (8)	C16—C15—H15	121.0 (17)
C17—P1—Ru1	123.10 (8)	C14—C15—H15	118.8 (17)
C23—O1—P1	119.18 (15)	C15—C16—C11	120.6 (3)
C6—C1—C2	117.0 (2)	C15—C16—H16	123.9 (18)
C6—C1—C7	121.6 (3)	C11—C16—H16	115.5 (18)
C2—C1—C7	121.4 (3)	C22—C17—C18	119.6 (2)
C6—C1—Ru1	70.10 (14)	C22—C17—P1	123.84 (18)
C2—C1—Ru1	71.82 (15)	C18—C17—P1	116.54 (19)
C7—C1—Ru1	130.0 (2)	C19—C18—C17	120.3 (3)
C3—C2—C1	122.3 (3)	C19—C18—H18	116.4 (17)
C3—C2—Ru1	72.54 (15)	C17—C18—H18	123.2 (17)
C1—C2—Ru1	70.73 (15)	C18—C19—C20	119.5 (3)
C3—C2—H2	122.3 (19)	C18—C19—H19	119.9 (19)
C1—C2—H2	114.8 (19)	C20—C19—H19	120.6 (19)
Ru1—C2—H2	123.0 (17)	C21—C20—C19	120.4 (3)
C2—C3—C4	120.5 (2)	C21—C20—H20	120.2 (19)
C2—C3—Ru1	71.44 (15)	C19—C20—H20	119.2 (19)
C4—C3—Ru1	71.21 (14)	C20—C21—C22	120.4 (3)
C2—C3—H3	119.4 (16)	C20—C21—H21	119.5 (19)
C4—C3—H3	119.8 (16)	C22—C21—H21	120.1 (19)
Ru1—C3—H3	125.2 (16)	C17—C22—C21	119.7 (2)
C5—C4—C3	118.0 (2)	C17—C22—H22	121.4 (15)
C5—C4—C8	123.3 (3)	C21—C22—H22	118.8 (15)
C3—C4—C8	118.7 (2)	O1—C23—C24	107.2 (2)
C5—C4—Ru1	70.18 (14)	O1—C23—H23A	105.7 (18)
C3—C4—Ru1	71.56 (14)	C24—C23—H23A	111.1 (18)
C8—C4—Ru1	130.35 (17)	O1—C23—H23B	109.4 (15)
C4—C5—C6	120.7 (2)	C24—C23—H23B	110.2 (15)
C4—C5—Ru1	72.94 (14)	H23A—C23—H23B	113 (2)
C6—C5—Ru1	70.22 (14)	C23—C24—H24A	109.7 (19)
C4—C5—H5	120.3 (16)	C23—C24—H24B	113 (2)
C6—C5—H5	118.6 (16)	H24A—C24—H24B	113 (3)
Ru1—C5—H5	123.4 (17)	C23—C24—H24C	107 (2)
C1—C6—C5	121.5 (2)	H24A—C24—H24C	105 (3)
C1—C6—Ru1	72.72 (15)	H24B—C24—H24C	109 (3)
C5—C6—Ru1	71.89 (14)		
			
C6—Ru1—P1—O1	−35.73 (10)	P1—Ru1—C4—C5	49.58 (16)
C5—Ru1—P1—O1	−73.63 (9)	Cl2—Ru1—C4—C5	132.70 (15)
C1—Ru1—P1—O1	−6.51 (10)	Cl1—Ru1—C4—C5	−139.44 (14)
C2—Ru1—P1—O1	−2.91 (19)	C6—Ru1—C4—C3	100.96 (17)
C4—Ru1—P1—O1	−100.89 (10)	C5—Ru1—C4—C3	130.2 (2)
C3—Ru1—P1—O1	−100.5 (2)	C1—Ru1—C4—C3	64.48 (16)
Cl2—Ru1—P1—O1	173.77 (7)	C2—Ru1—C4—C3	28.07 (15)
Cl1—Ru1—P1—O1	83.79 (7)	P1—Ru1—C4—C3	179.78 (13)
C6—Ru1—P1—C11	74.33 (11)	Cl2—Ru1—C4—C3	−97.10 (14)
C5—Ru1—P1—C11	36.43 (11)	Cl1—Ru1—C4—C3	−9.2 (2)
C1—Ru1—P1—C11	103.55 (12)	C6—Ru1—C4—C8	−146.6 (3)
C2—Ru1—P1—C11	107.15 (19)	C5—Ru1—C4—C8	−117.4 (3)
C4—Ru1—P1—C11	9.17 (11)	C1—Ru1—C4—C8	176.9 (3)
C3—Ru1—P1—C11	9.5 (2)	C2—Ru1—C4—C8	140.5 (3)
Cl2—Ru1—P1—C11	−76.17 (9)	C3—Ru1—C4—C8	112.4 (3)
Cl1—Ru1—P1—C11	−166.15 (9)	P1—Ru1—C4—C8	−67.8 (3)
C6—Ru1—P1—C17	−163.51 (12)	Cl2—Ru1—C4—C8	15.3 (2)
C5—Ru1—P1—C17	158.59 (11)	Cl1—Ru1—C4—C8	103.2 (3)
C1—Ru1—P1—C17	−134.29 (12)	C3—C4—C5—C6	−1.5 (3)
C2—Ru1—P1—C17	−130.70 (19)	C8—C4—C5—C6	179.5 (2)
C4—Ru1—P1—C17	131.33 (11)	Ru1—C4—C5—C6	53.6 (2)
C3—Ru1—P1—C17	131.7 (2)	C3—C4—C5—Ru1	−55.1 (2)
Cl2—Ru1—P1—C17	45.98 (9)	C8—C4—C5—Ru1	125.9 (2)
Cl1—Ru1—P1—C17	−43.99 (9)	C6—Ru1—C5—C4	132.7 (2)
C11—P1—O1—C23	176.00 (19)	C1—Ru1—C5—C4	103.97 (17)
C17—P1—O1—C23	71.5 (2)	C2—Ru1—C5—C4	66.18 (16)
Ru1—P1—O1—C23	−65.57 (19)	C3—Ru1—C5—C4	30.32 (15)
C5—Ru1—C1—C6	29.19 (15)	P1—Ru1—C5—C4	−138.82 (14)
C2—Ru1—C1—C6	128.8 (2)	Cl2—Ru1—C5—C4	−52.87 (16)
C4—Ru1—C1—C6	65.40 (16)	Cl1—Ru1—C5—C4	120.15 (18)
C3—Ru1—C1—C6	101.78 (17)	C1—Ru1—C5—C6	−28.68 (15)
P1—Ru1—C1—C6	−53.82 (16)	C2—Ru1—C5—C6	−66.47 (16)
Cl2—Ru1—C1—C6	125.43 (19)	C4—Ru1—C5—C6	−132.7 (2)
Cl1—Ru1—C1—C6	−144.56 (15)	C3—Ru1—C5—C6	−102.33 (17)
C6—Ru1—C1—C2	−128.8 (2)	P1—Ru1—C5—C6	88.53 (14)
C5—Ru1—C1—C2	−99.58 (17)	Cl2—Ru1—C5—C6	174.48 (13)
C4—Ru1—C1—C2	−63.37 (17)	Cl1—Ru1—C5—C6	−12.5 (3)
C3—Ru1—C1—C2	−26.99 (16)	C2—C1—C6—C5	1.1 (4)
P1—Ru1—C1—C2	177.41 (14)	C7—C1—C6—C5	179.5 (3)
Cl2—Ru1—C1—C2	−3.3 (3)	Ru1—C1—C6—C5	−55.1 (2)
Cl1—Ru1—C1—C2	86.67 (16)	C2—C1—C6—Ru1	56.2 (2)
C6—Ru1—C1—C7	115.1 (4)	C7—C1—C6—Ru1	−125.4 (3)
C5—Ru1—C1—C7	144.3 (3)	C4—C5—C6—C1	0.6 (4)
C2—Ru1—C1—C7	−116.1 (4)	Ru1—C5—C6—C1	55.5 (2)
C4—Ru1—C1—C7	−179.5 (3)	C4—C5—C6—Ru1	−54.8 (2)
C3—Ru1—C1—C7	−143.1 (3)	C5—Ru1—C6—C1	−132.7 (2)
P1—Ru1—C1—C7	61.3 (3)	C2—Ru1—C6—C1	−31.19 (16)
Cl2—Ru1—C1—C7	−119.4 (3)	C4—Ru1—C6—C1	−104.13 (17)
Cl1—Ru1—C1—C7	−29.4 (3)	C3—Ru1—C6—C1	−66.83 (16)
C6—C1—C2—C3	−2.0 (4)	P1—Ru1—C6—C1	133.78 (15)
C7—C1—C2—C3	179.6 (3)	Cl2—Ru1—C6—C1	−143.00 (14)
Ru1—C1—C2—C3	53.3 (2)	Cl1—Ru1—C6—C1	41.76 (17)
C6—C1—C2—Ru1	−55.3 (2)	C1—Ru1—C6—C5	132.7 (2)
C7—C1—C2—Ru1	126.3 (3)	C2—Ru1—C6—C5	101.47 (17)
C6—Ru1—C2—C3	−103.75 (19)	C4—Ru1—C6—C5	28.53 (15)
C5—Ru1—C2—C3	−65.80 (17)	C3—Ru1—C6—C5	65.83 (16)
C1—Ru1—C2—C3	−134.7 (3)	P1—Ru1—C6—C5	−93.57 (14)
C4—Ru1—C2—C3	−28.96 (16)	Cl2—Ru1—C6—C5	−10.3 (2)
P1—Ru1—C2—C3	−140.02 (16)	Cl1—Ru1—C6—C5	174.42 (12)
Cl2—Ru1—C2—C3	43.85 (19)	C5—C4—C8—C10	−9.4 (4)
Cl1—Ru1—C2—C3	132.65 (16)	C3—C4—C8—C10	171.6 (3)
C6—Ru1—C2—C1	30.98 (16)	Ru1—C4—C8—C10	82.1 (4)
C5—Ru1—C2—C1	68.93 (16)	C5—C4—C8—C9	116.6 (3)
C4—Ru1—C2—C1	105.77 (18)	C3—C4—C8—C9	−62.3 (4)
C3—Ru1—C2—C1	134.7 (3)	Ru1—C4—C8—C9	−151.9 (3)
P1—Ru1—C2—C1	−5.3 (3)	O1—P1—C11—C12	27.2 (2)
Cl2—Ru1—C2—C1	178.57 (13)	C17—P1—C11—C12	132.9 (2)
Cl1—Ru1—C2—C1	−92.62 (15)	Ru1—P1—C11—C12	−93.6 (2)
C1—C2—C3—C4	1.1 (4)	O1—P1—C11—C16	−155.1 (2)
Ru1—C2—C3—C4	53.6 (2)	C17—P1—C11—C16	−49.3 (2)
C1—C2—C3—Ru1	−52.5 (2)	Ru1—P1—C11—C16	84.2 (2)
C6—Ru1—C3—C2	65.11 (18)	C16—C11—C12—C13	−0.7 (4)
C5—Ru1—C3—C2	102.84 (18)	P1—C11—C12—C13	177.0 (2)
C1—Ru1—C3—C2	27.99 (17)	C11—C12—C13—C14	0.6 (4)
C4—Ru1—C3—C2	132.9 (2)	C12—C13—C14—C15	−0.2 (5)
P1—Ru1—C3—C2	132.40 (19)	C13—C14—C15—C16	−0.1 (5)
Cl2—Ru1—C3—C2	−143.59 (16)	C14—C15—C16—C11	−0.1 (4)
Cl1—Ru1—C3—C2	−52.27 (18)	C12—C11—C16—C15	0.5 (4)
C6—Ru1—C3—C4	−67.78 (15)	P1—C11—C16—C15	−177.3 (2)
C5—Ru1—C3—C4	−30.05 (14)	O1—P1—C17—C22	−128.6 (2)
C1—Ru1—C3—C4	−104.90 (17)	C11—P1—C17—C22	130.4 (2)
C2—Ru1—C3—C4	−132.9 (2)	Ru1—P1—C17—C22	3.9 (2)
P1—Ru1—C3—C4	−0.5 (3)	O1—P1—C17—C18	53.3 (2)
Cl2—Ru1—C3—C4	83.53 (14)	C11—P1—C17—C18	−47.7 (2)
Cl1—Ru1—C3—C4	174.85 (12)	Ru1—P1—C17—C18	−174.27 (16)
C2—C3—C4—C5	0.7 (4)	C22—C17—C18—C19	−1.5 (4)
Ru1—C3—C4—C5	54.4 (2)	P1—C17—C18—C19	176.7 (2)
C2—C3—C4—C8	179.7 (2)	C17—C18—C19—C20	1.0 (5)
Ru1—C3—C4—C8	−126.5 (2)	C18—C19—C20—C21	0.3 (5)
C2—C3—C4—Ru1	−53.8 (2)	C19—C20—C21—C22	−1.1 (5)
C6—Ru1—C4—C5	−29.25 (15)	C18—C17—C22—C21	0.8 (4)
C1—Ru1—C4—C5	−65.72 (16)	P1—C17—C22—C21	−177.3 (2)
C2—Ru1—C4—C5	−102.14 (17)	C20—C21—C22—C17	0.5 (4)
C3—Ru1—C4—C5	−130.2 (2)	P1—O1—C23—C24	163.4 (2)

### Selected geometric parameters (Å, °) for the title compound and related compounds

Σ angles = sum of P—Ru—Cl1, P—Ru—Cl2, and Cl1—Ru—Cl2 angles.

**Table:**

	Title compound	[Ru(η^6^-p-cymene)Cl_2_PPhOEt_2_]*^a^*	[Ru(η^6^-p-cymene)Cl_2_PPh_3_]*^b^*
Ru—P	2.3147 (6)	2.2807 (7)	2.3438 (6)
Ru—Cl1	2.4124 (6)	2.4171 (7)	2.4154 (6)
Ru—Cl2	2.3992 (6)	2.4038 (7)	2.4151 (6)
Ru—C(av)	2.217 (1)	2.218 (4)	2.218 (2)
P—Ru – Cl1	90.67 (2)	87.59 (2)	87.094 (19)
P—Ru – Cl2	84.75 (2)	87.89 (2)	90.27 (2)
Cl1—Ru – Cl2	90.04 (2)	88.81 (2)	88.41 (2)
Σ angles	265.46	264.29	265.77

Notes: (*a*) Albertin *et al.* (2010); (*b*) Elsegood *et al.* (2006).
